# Phenotypic expansion of the *BPTF*‐related neurodevelopmental disorder with dysmorphic facies and distal limb anomalies

**DOI:** 10.1002/ajmg.a.62102

**Published:** 2021-01-31

**Authors:** Kevin E. Glinton, Anna C. E. Hurst, Kevin M. Bowling, Ingrid Cristian, Devon Haynes, Dusit Adstamongkonkul, Oskar Schnappauf, David B. Beck, Carole Brewer, Aditi Shah Parikh, Deepali N. Shinde, Alan Donaldson, Ariel Brautbar, Saskia Koene, Arie van Haeringen, Amélie Piton, Yline Capri, Margherita Furlan, Elena Gardella, Rikke Steensbjerre Møller, Irma van de Beek, Linda Zuurbier, Phillis Lakeman, Allan Bayat, Julian Martinez, Rebecca Signer, Pernille M. Torring, Morten Buch Engelund, Karen W. Gripp, Louise Amlie‐Wolf, Lindsay B. Henderson, Alina T. Midro, Eugeniusz Tarasów, Beata Stasiewicz‐Jarocka, Diana Moskal‐Jasinska, Paul Vos, Felix Boschann, Corinna Stoltenburg, Oliver Puk, Inger‐Lise Mero, Kristine Lossius, Cyril Mignot, Boris Keren, Johanna C. Acosta Guio, Ignacio Briceño, Alberto Gomez, Yaping Yang, Pawel Stankiewicz

**Affiliations:** ^1^ Department of Molecular & Human Genetics Baylor College of Medicine, One Baylor Plaza Houston Texas USA; ^2^ Department of Genetics University of Alabama at Birmingham Birmingham Alabama USA; ^3^ HudsonAlpha Institute for Biotechnology Huntsville Alabama USA; ^4^ Division of Genetics Arnold Palmer Hospital for Children – Orlando Health Orlando Florida USA; ^5^ CoxHealth CoxHealth Pediatric Specialties Springfield Missouri USA; ^6^ University of Missouri School of Medicine Springfield Clinical Campus Springfield Missouri USA; ^7^ National Human Genome Research Institute National Institutes of Health Bethesda Maryland USA; ^8^ Peninsula Clinical Genetics Service Royal Devon and Exeter NHS Foundation Trust Exeter United Kingdom; ^9^ Center for Human Genetics University Hospitals Cleveland Medical Center and Case Western Reserve University Cleveland Ohio USA; ^10^ Department of Clinical Genomics Ambry Genetics Aliso Viejo California USA; ^11^ Clinical Genetics University Hospitals Bristol NHS Foundation Trust Bristol United Kingdom; ^12^ Medical Genetics Department Cook Children's Hospital Fort Worth Texas USA; ^13^ Department of Clinical Genetics Leiden University Medical Center Leiden The Netherlands; ^14^ Unité de Génétique Moléculaire Strasbourg University Hospital 1 place de l'Hôpital Strasbourg Cedex France; ^15^ Service de Génétique Clinique CHU Robert Debré Paris Cedex France; ^16^ Danish Epilepsy Centre Dianalund Denmark; ^17^ University of Southern Denmark Odense Denmark; ^18^ Amsterdam UMC University of Amsterdam, Department of Clinical Genetics Amsterdam the Netherlands; ^19^ Department of Pediatrics University Hospital of Hvidovre Copenhagen Denmark; ^20^ Departments of Human Genetics, Pediatrics and Psychiatry David Geffen School of Medicine at UCLA California Los Angeles USA; ^21^ Department of Clinical Genetics Odense University Hospital Odense Denmark; ^22^ Division of Medical Genetics Nemours/Alfred I. duPont Hospital for Children Wilmington Delaware USA; ^23^ GeneDx Gaithersburg Maryland USA; ^24^ Department of Clinical Genetics Medical University, Białystok, 15‐089 Białystok Poland; ^25^ Department of Radiology Medical University Bialystok Poland; ^26^ Department of Clinical Phonoaudiology and Speech Therapy Medical University, Białystok Białystok Poland; ^27^ Department of Pediatrics Haga Teaching Hospital, Juliana Children's Hospital The Hague The Netherlands; ^28^ Institut für Medizinische Genetik und Humangenetik Charité ‐ Universitätsmedizin Berlin, Corporate Member of Freie Universität Berlin, Humboldt Universität zu Berlin, and Berlin Institute of Health Berlin Germany; ^29^ Department of Neuropaediatrics Charité – Berlin University of Medicine Berlin Germany; ^30^ Praxis für Humangenetik Tuebingen Department of Genetic Diagnostics Tuebingen Germany; ^31^ Department of Medical Genetics Oslo University Hospital Norway; ^32^ Department of Pediatric and Adolescent Medicine Akershus University Hospital Norway; ^33^ APHP‐Sorbonne Université Département de Génétique, Hôpital Trousseau et Groupe Hospitalier Pitié‐Salpêtrière Paris France; ^34^ Department of Genetics APHP, Pitié‐Salpêtrière University Hospital Paris France; ^35^ Especialista en Genética Médica Instituto de Ortopedia Infantil Roosevelt Bogotá Cundinamarca Colombia; ^36^ Instituto de Genética Humana Facultad de Medicina, Pontificia Universidad Javeriana Bogotá DC Colombia; ^37^ AiLife Diagnostics Country Place Pkwy Suite 100 Pearland Texas USA

**Keywords:** chromatin remodeling, epilepsy, microcephaly

## Abstract

Neurodevelopmental disorder with dysmorphic facies and distal limb anomalies (NEDDFL), defined primarily by developmental delay/intellectual disability, speech delay, postnatal microcephaly, and dysmorphic features, is a syndrome resulting from heterozygous variants in the dosage‐sensitive bromodomain PHD finger chromatin remodeler transcription factor *BPTF* gene. To date, only 11 individuals with NEDDFL due to de novo *BPTF* variants have been described. To expand the NEDDFL phenotypic spectrum, we describe the clinical features in 25 novel individuals with 20 distinct, clinically relevant variants in *BPTF*, including four individuals with inherited changes in *BPTF*. In addition to the previously described features, individuals in this cohort exhibited mild brain abnormalities, seizures, scoliosis, and a variety of ophthalmologic complications. These results further support the broad and multi‐faceted complications due to haploinsufficiency of *BPTF*.

## 1 INTRODUCTION

The bromodomain PHD finger transcription factor (BPTF) is the largest subunit of the nucleosome remodeling factor (NURF), a member of the imitation switch (ISWI) chromatin remodeling complex family (Bartholomew, 2014). NURF complexes have been shown to catalyze ATP‐dependent nucleosome sliding and facilitate transcription of chromatin (Hamiche et al., 1999), thereby regulating various genes and helping govern higher‐order chromatin structures. BPTF in particular facilitates this interaction by the preferential binding of its plant homeodomain (PHD) finger to the tails of the trimethylated lysine 4 of histone H3 (H3K4me3) and binding of its bromodomain to the acetylated lysine 16 of histone H4 (H4K16ac) (Filippakopoulos et al., 2012; Wysocka et al., 2006). Animal studies have demonstrated the essential role of *Bptf* in the differentiation of the primary germ layers and establishment of the embryonal anterior–posterior axis (Landry et al., 2008). Recent unpublished studies showed that Emx1‐Cre driven inactivation of *Bptf* in the developing mouse forebrain results in animals with a severe reduction in cortical mass with little to no effect in heterozygous animals (David Picketts, personal communication). Human studies have detailed extensively the role of *BPTF* in the development of several malignancies (Dai et al., 2015; Green et al., 2020; Lee et al., 2016; Richart et al., 2016; Zhao et al., 2019) and in T‐cell homeostasis and function (Mayes et al., 2016; Wu et al., 2016).

The significance of constitutional, pathogenic variants in *BPTF* was reported in 2017, when the first 10 individuals with heterozygous, pathogenic single nucleotide or copy‐number deletion variants were described (Stankiewicz et al., 2017). Haploinsufficiency of *BPTF* in humans leads to the clinical entity known as neurodevelopmental disorder with dysmorphic facies and distal limb anomalies (NEDDFL, MIM#617755). The syndrome consists primarily of developmental delay (DD)/intellectual disability (ID), speech delay, postnatal microcephaly, and dysmorphic features. The disorder remains, however, incompletely differentiated with few further individuals described in the medical literature (Deciphering Developmental Disorders, 2015, 2017; Midro et al., 2019; Popp et al., 2017). All individuals identified thus far, have carried heterozygous de novo changes with mostly copy‐number variant (CNV) deletions and frameshift, or nonsense single‐nucleotide variants (SNVs). Based on these publications, it has remained unclear whether pathogenic variants may be inherited and whether deleterious variants are fully or incompletely penetrant.

Here, we describe 25 novel patients with NEDDFL due to 20 distinct variants in *BPTF*, including, for the first time, four patients found to have inherited a causative variant from their apparently non‐mosaic, affected parents. The study provides additional insight into the phenotypic features of this disorder and expands on our knowledge of the inheritance and penetrance of this still new disorder.

## 2 MATERIAL AND METHODS

### 2.1 Editorial policies and ethical considerations

This study was conducted in accordance with the ethical standards of the Baylor College of Medicine Committee on Human Research.

### 2.2 Patients and recruitment

Subjects were identified and recruited either through their treating clinicians, self‐referral, or GeneMatcher (Sobreira et al., 2015). Molecular testing results from exome sequencing, chromosomal microarray, or next‐generation sequencing panel were submitted by patients' healthcare providers (See “Supplementary Materials” for details of each test's composition). Basic medical information including birth parameters, developmental histories, and physical examinations were collected from healthcare providers and/or patient families. Percentiles and z‐scores for height, weight and head circumference were calculated based on the Centers for Disease Control and Prevention (CDC) growth charts using the PediTools (https://peditools.org) (Chou et al., 2020) or SimulConsult (https://simulconsult.com/resources/measurement.html) online calculators. All photographs submitted are used with the written consent of patients or guardians as appropriate. In the case of patients lost to follow‐up or for whom no clinical data were available, only basic demographic and molecular findings are reported.

## 3 RESULTS

### 3.1 Overview

We describe 26 individuals from 21 families, including four cases of familial or inherited variants, including one (Patient 22) previously reported (Midro et al., 1993, 2019). Patient 1 inherited her variant from her mother (Patient 2), Patient 9 was found to have inherited his variant from his mother (Patient 10), Patient 13 is the father of Patient 12, and Patient 16 is the mother of Patient 15. The affected individuals included 14 males and 11 females (no demographic data were provided for Patient 11), aged 23 months–55 years at last clinical assessment. Common features included DD and/or ID (23/26, 88%), speech delay (22/26, 85%), head circumference less than the third percentile for age (11/26, 42%), motor delay (18/26, 69%), hypotonia (10/26, 38%), and dysmorphic features (20/26, 77%).

### 3.2 Molecular findings

**FIGURE 1:**
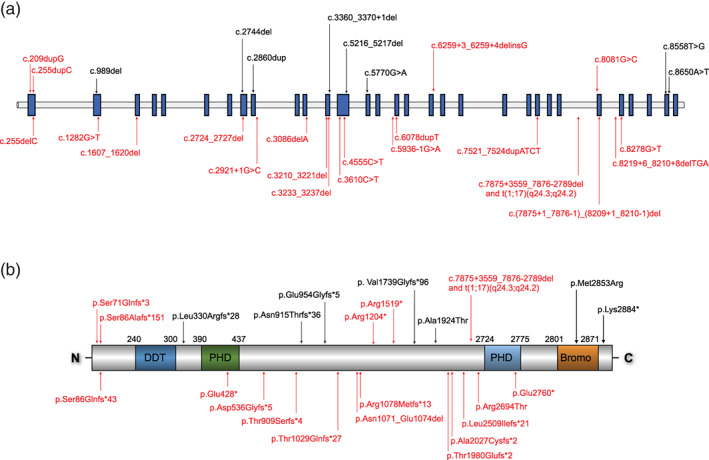
Schematic of variants in the (a) *BPTF* gene and (b) BPTF protein. Variants in black represent variants reported previously. Variants in red represent variants described in the present study [Color figure can be viewed at wileyonlinelibrary.com]

Our analysis identified 20 novel, distinct variants in *BPTF* (Table 1) distributed throughout the gene (Figure 1), including 14 de novo and four inherited. Unique exonic variants include nine frameshift, four nonsense, three splicing, two in‐frame deletions, one missense, and one single exon truncating deletion (Tables 1 and S1). We also describe one previously published chromosomal translocation and CNV deletion disrupting *BPTF* (Patient 22) (Midro et al., 1993; Midro et al., 2019). Variants were interpreted as pathogenic (11), likely pathogenic (7), or VUS (2) based on the current ACMG criteria. There does not appear to be a predominant genotype–phenotype correlation within our cohort though it is worth noting that manifestations were milder in the lone individual with a missense variant (Patient 23) when compared to the rest of the cohort.

**TABLE 1:** Birth and current growth parameters of patients with *BPTF* variants (isoform NM_004459.6)

Patient	Variant	Variant type	Inheritance	Sex	Age	Gestational age (weeks)	APGAR scores	Weight at birth (kg) (Z score)	Length at birth (cm) (Z score)	Head circumference at birth (cm) (Z score)	Age at last evaluation	Weight (kg) (Z score)	Length (cm) (Z score)	Head circumference (cm) (Z score)
1	c.209dupG (p.Ser71Glnfs*3)	Frameshift	Maternal	F	23 months	38 weeks	N/A	2.070 (−2.46)	N/A	N/A	23 months	10.6 (−1.12)	84 (−0.27)	44.5 (−2.01)
2 *(mother of Patient 1)*	c.209dupG (p.Ser71Glnfs*3)	Frameshift	N/A	F	N/A	Term	N/A	N/A	N/A	N/A	N/A	N/A	N/A	N/A
3	c.255delC (p.Ser86Alafs*151)	Frameshift	de novo	F	22 years	N/A	N/A	N/A	N/A	N/A	22 years	35.4 (−3.30)	127 (−5.54)	N/A
4	c.255delC (p.Ser86Alafs*151)	Frameshift	de novo	M	7 years 1 month	N/A	N/A	2.635 (−1.57)	49.53 (−0.19)	N/A	7 years 1 month	18.7 (−1.70)	119.75 (−1.03)	N/A
5	c.255dupC (p.Ser86Glnfs*43)	Frameshift	de novo	F	10 years	37 weeks	N/A	2.270 (−2.13)	46.36 (−1.27)	32.385 (−1.55)	10 years	26.36 (−1.25	136.5 (−0.22)	46.5 (−4.47)
6	c.1282G > T (p.Glu428*)	Nonsense	de novo	M	13 years	39 weeks	N/A	3.310 (−0.40)	53.40 (+1.30)	N/A	13 years	106.7 (+3.25)	168 (+1.50)	53.4 (−0.54)
7	c.1607_1620del (p.Asp536Glyfs*5)	Frameshift	N/A	M	12 years 8 months	35 weeks	10, 10	1.720 (−2.64)	N/A	31 (−2.04)	12 years 8 months	39.5 (−0.55)	153 (−0.07)	39.5 (−9.61)
8	c.2724_2727del (p.Thr909Serfs*4)	Frameshift	de novo	F	2 years	39 weeks 3 days	5, 8	2.470 (−1.78)	49.00 (−0.12)	30 (−3.42)	2 years	9.5 (−2.42)	83.1 (−0.54)	45.1 (−1.67)
9	c.2921+1G > C	Splicing	Maternal	M	14 years 3 months	38 weeks	N/A	2.830 (−1.20)	N/A	N/A	14 years 3 months	36.6 (−2.12)	160.5 (−0.63)	51.1 (−2.33)
10 *(mother of Patient 9)*	c.2921+1G > C	Splicing	N/A	F	N/A	N/A	N/A	N/A	N/A	N/A	N/A	N/A	160 (−0.50)	49.4 (−4.70)
11	c.3085delA (p.Thr1029Glnfs*27)	Frameshift	de novo	N/A	N/A	N/A	N/A	N/A	N/A	N/A	N/A	N/A	N/A	N/A
12	c.3210_3221del (p.Asn1071Glu1074del)	In‐frame deletion	Paternal	M	11 years 1 month	Term	N/A	3.690 (+0.30)	N/A	N/A	11 years 1 month	25.6 (−2.20)	131.7 (−1.79)	52 (0.90)
13 *(father of Patient 12)*	c.3210_3221del (p.Asn1071Glu1074del)	In‐frame deletion	N/A	M	55 years	N/A	N/A	N/A	N/A	N/A	55 years	N/A	N/A	N/A
14	c.3233_3237del (p.Arg1078Metfs*13)	Frameshift	N/A	M	8 years	42 weeks	N/A	2.75 (−1.32)	N/A	N/A	8 years	16.4 (−3.94)	112 (−2.85)	48 (−3.11)
15	c.3610C > T (p.Arg1204*)	Nonsense	Maternal	M	4 years 6 months	Term	10, 10	2.740 (−1.33)	46.50 (−1.30)	31 (−2.04)	4 years 6 months	14.7 (−1.41)	102 (−0.81)	47 (−2.60)
16 *(mother of patient 15)*	c.3610C > T (p.Arg1204*)	Nonsense	N/A	F	N/A	N/A	N/A	N/A	N/A	N/A	N/A	N/A	N/A	N/A
17	c.4555C > T (p.Arg1519*)	Nonsense	de novo	F	9 years	40 weeks 1 day	N/A	2.500 (−2.13)	47.00 (−1.12)	N/A	9 years	20 (−2.42)	131 (−0.31)	49.5 (−2.39)
18	c.5936‐1G > A (p.Thr1980Glufs25)	Splicing	de novo	M	8 years 8 months	36 weeks 3 days	9, 10	2.000 (−1.43)	N/A	N/A	8 years 8 months	18 (−3.57)	118.7 (−2.25)	48.5 (−3.01)
19	c.6078dupT (p.Ala2027Cysfs*2)	Frameshift	de novo	M	12 years	40 weeks 1 day	N/A	3.320 (−0.38)	48.00 (−0.75)	N/A	12 years	31.9 (−1.34)	149.5 (+0.06)	50 (−2.55)
20	c.6259+3_6259+4delinsG	Splicing	de novo	F	9 years	38 weeks	10	2.656 (−1.45)	56.00 (+2.35)	32 (−1.83)	9 years	18.9 (−2.88)	122 (−1.82)	49.5 (−1.97)
21	c.7521_7524dupATCT (p.Leu2509Ilefs*21)	Frameshift	de novo	M	10 years	37 weeks	2, 9	2.770 (−1.29)	41.00 (−3.30)	32 (−1.70)	10 years	30.8 (−0.21)	122.9 (−2.46)	51.3 (−1.17)
22^a^	c.7875+3559_7876‐2789del and t(1;17)(q24.3;q24.2)	Gene disruption/deletion	de novo	M	35 years	Term	N/A	2.550 (−1.61)	49.00 (−0.37)	30 (−2.35)	8 years	18 (−2.91)	116 (−2.11)	49 (−2.39)
23	c.8081G > C (p.Arg2694Thr)	Missense	N/A^b^	M	9 years	38 weeks	N/A	3.800 (+0.52)	N/A	N/A	9 years	20 (−2.75)	110 (−4.03)	50 (−1.96)
24	c.(7875+1_7876–1)_(8209+1_8210‐1)del	Exon deletion, Frameshift	de novo	F	12 years 3 months	40 weeks	9, 10, 10	2.700 (−1.37)	49.00 (−0.12)	33 (−1.11)	12 years 3 months	27 (−2.77)	139 (−1.88)	51 (−1.71)
25	c.8210+6_8210+8del	In‐frame deletion	de novo	F	2 years	40 weeks 4 days	6, 8, 9	3.120 (−0.71)	50.00 (+0.09)	34 (−0.63)	1 years 6 months	11 (+0.01)	86 (+1.79)	46 (−0.39)
26	c.8278G > T (p.Glu2760*)	Nonsense	de novo	M	11 years	32 weeks	8, 10	2.480 (−1.71)	46.5 (−1.30)	33 (−1.32)	11 years	39 (+0.42)	153 (+1.33)	53.5 (0.21)

Abbreviation: N/A, not available or not reported.

^a^ Previously reported (Midro et al., 1993, 2019).

^b^ Parental samples were negative by Sanger sequencing. Paternity and maternity were not confirmed.

### 3.3 Clinical findings

#### 3.3.1 Birth history and growth parameters

Most individuals in our cohort were born at term (median 38, range 36–42 weeks) following reportedly uncomplicated pregnancies (Table 1). When available, APGAR scores were within normal limits (median at 1 min = 9 (range 2–10); median at 5 min = 10 (range 8–10)). Available birth parameters showed that 13 individuals were small for gestational age (i.e., birth weight less than the 10th percentile) though only one patient had a length below the third percentile. Of the individuals with measurements available, 4/10 (40%) had a head circumference less than the third percentile for gestational age at birth.

At the time of their most recent clinical assessment, 5/20 (25%) individuals exhibited short stature for age and 10/19 (53%) individuals had decreased weight for age (based on measurements less than the third percentiles for height and weight respectively). Though head circumferences were not provided for every individual, consistent with previous observations (Stankiewicz et al., 2017), 12/20 (60%) individuals demonstrated a head circumference less than the third percentile for age.

#### 3.3.2 Development and neurologic findings

In our cohort, 23/26 (88%) patients demonstrated some form of DD, ranging from severe to only mild delays/deficits (Table 2). In the cases of Patient 12 and Patient 13 (proband and father, respectively), only mild deficits were noted and Patient 13 had been able to obtain full time employment and attain full independence in society. A history of speech delay was described in 22/26 (85%) of individuals, whereas 18/26 (69%) individuals reported a history of motor delays and 10/26 (38%) a history of hypotonia. In addition, six individuals reported a history of seizures and EEG abnormalities: Patient 6 has been treated with sodium valproate, Patients 1 and 17 have been treated with levetiracetam and Patient 21 eventually required vagal nerve stimulator placement for seizure control. In both Patients 24 and 26, only electrographic abnormalities were seen and not clinical seizures. Magnetic resonance imaging (MRI) studies were obtained for 13/26 patients and were found to be normal in 8/13 cases. The remaining five studies demonstrated mild structural abnormalities only **(**Table 2 **)**.

**TABLE 2:** Neurodevelopmental and dysmorphic features of patients with variants in *BPTF*

ID	Variant	Sex	Age	DD/ID	Speech delay	Motor delay	Hypotonia	Autism	ADHD	Dysmorphic features	Eye anomalies	Skeletal anomalies	Brain anomalies	Seizures and/or EEG abnormalities	Other comments
Patient 1	c.209dupG (p.Ser71Glnfs*3)	F	23 months	+	+	+	+	N/A	N/A	Bilateral epicanthal folds, slightly short palpebral fissures, tubular nose, flat cheekbones, fetal fingertip pads, mild 2–3 syndactyly of the feet, borderline low tone	>Bilateral myopia with astigmatism	N/A	Minimal asymmetry of the temporal horn of the right lateral ventricle; no other intracranial pathology.	Diagnosed with epilepsy and has generalized tonic–clonic seizures as well as staring spells.	History of feeding difficulties with oral aversion, GERD, poor weight gain and constipation. Diagnosed with global developmental delay (DQ =
Patient 2 *(mother of Patient 1)*	c.209dupG (p.Ser71Glnfs*3)	F	N/A	+	+	+	N/A	N/A	N/A	Short and narrow palpebral fissures, prominent nasal ridge, broad great toes.	Exotropia	N/A	N/A	N/A	SGA at delivery, delayed walking and talking; described as a “poor eater.” Difficulties in school and repeated kindergarten twice. Intellectual ability in the “borderline range” per school testing. Is generally able to manage her own affairs but needs assistance with some tasks like paying bills.
Patient 3	c.255delC (p.Ser86Alafs*151)	F	22 years	+	+	+	+	+	+	Prominent nasal ridge, thin upper lip	N/A	N/A	N/A	N/A	Hypothyroidism, poor weight gain and decreased growth velocity; anxiety, depression; recurrent oral ulcers. Required inclusion classroom with 40% special education; now resides in a group home. First words said on time but did not progress normally. Now able to speak in sentences and is able to write.
Patient 4	c.255delC (p.Ser86Alafs*151)	M	7 years 1 month	+	+	N/A	+	N/A	N/A	N/A	None	N/A	N/A	N/A	N/A
Patient 5	c.255dupC (p.Ser86Glnfs*43)	F	10 years	+	+	+	−	N/A	N/A	Triangular‐shaped face, café au lait macules (left medial calf 2.5 cm × 1 cm, left ASIS 7 mm, right anterior chest‐abdomen 5 mm, left anterior chest 3 mm, right lateral thigh 1 cm × 1.5 cm, left lateral hip 7 mm)	Intermittent exotropia, emmetropia	S‐shaped scoliosis of the thoracolumbar spine; mild kyphotic deformity of the thoracic spine; mild anterior wedging of mid‐thoracic vertebra; length discrepancy of arms (right arm longer than left arm by about 5–7 mm)	Normal brain MRI	N/A	Poor weight gain/failure to thrive; in utero growth retardation; increased muscle fatigability and abnormal fingers usage; mild elevation of LDL and mildly depressed HDL
Patient 6	c.1282G > T (p.Glu428*)	M	13 years	+ (mild)	+ (mild)	+	+	N/A	N/A	Long nasal bridge, retrognathia, high palate	N/A	N/A	Small venous anomaly of right front; no other abnormality	Epilepsy requiring sodium valproate	Recurrent fevers, oral ulcers, skin rashes, initially requiring treatment now improved; dysphonia
Patient 7	c.1607_1620del (p.Asp536Glyfs*5)	M	12 years 8 months	+ (mild)	+	−	−	N/A	+	Metopic ridge, brachycephaly, mild micrognathia, synophrys, apparently long tongue, cutis marmorata; square finger tips, moderate soft tissue syndactyly of toes 2 and 3, broad great toes and thumbs, wide sandal gap deformities	Left convergent squint ‐ resolved	Very mild scoliosis, delayed bone age (3 months at 18 months; normalized at age 8), metopic synostosis; prominent lumbar lordosis	Normal brain MRI	N/A	Constipation, hypertonia (~age 12), with brisk reflexes and clonus, recurrent facial nerve palsy (age 5‐6 years); pupillary hippus, early dental caries, cutis marmorata and poor peripheral circulation
Patient 8	c.2724_2727del (p.Thr909Serfs*4)	F	2 years	+	+	+	−	N/A	N/A	Epicanthal folds, prominent pointed helices with prominent antihelices; prominent nasal root, mild bulbous nasal tip	Astigmatism, myopia, right eye exotropia	Broad great toes	Hypotelorism; otherwise unremarkable MRI of the brain	N/A	Little subcutaneous fat, feeding issues
Patient 9	c.2921+1G > C	M	14 years 3 months	+ (mild)	+ (mild)	+	+	N/A	N/A	Thin upper lip, wide mouth.	None	None	N/A	N/A	Initial failure to thrive requiring gastrostomy; recurrent bacterial infections as a young child, requiring regular immunoglobulin infusions until age 4 y. First walked at 2 years. In mainstream school with statement of special educational needs.
Patient 10 *(mother of Patient 9)*	c.2921+1G > C	F	N/A	−	−	N/A	N/A	N/A	N/A	N/A	N/A	N/A	N/A	N/A	N/A
Patient 11	c.3085delA (p.Thr1029Glnfs*27)	N/A	N/A	+	N/A	N/A	N/A	N/A	N/A	Prominent nasal tip, high arched palate, brachycephaly, micrognathia, short stature.	“Weak” vision reported	Radioulnar stenosis	N/A	N/A	N/A
Patient 12	c.3210_3221del (p.Asn1071_Glu1074del)	M	11 years 1 month	+ (mild)	+	+	N/A	N/A	N/A	Right microtia with over‐folded helix, epicanthal folds, upslanting palpebral fissures, high palate, micrognathia, bulbous nasal tip; small body habitus (including hands and feet), fine hair on the back, one hyperpigmented and one hypopigmented macule	N/A	Right thumb is smaller than the left (4 cm vs. 3.9 cm); second toe is bent in a flexed position, but not contracted bilaterally; broad halluces	Normal brain MRI	N/A	Right microtia also seen in mother without BPTF variant
Patient 13 *(father of Patient 12)*	c.3210_3221del (p.Asn1071_Glu1074del)	M	55 years	+ (mild)	N/A	N/A	N/A	N/A	N/A	N/A	N/A	N/A	N/A	N/A	Mild delays in school, but employed.
Patient 14	c.3233_3237del (p.Arg1078Metfs*13)	M	8 years	+	+	+	−	+	N/A	Broad halluces, small chin, prominent columella, thin upper lip.	N/A	Delayed bone age (8 m)	N/A	N/A	IQ 60. Started talking around the age of 3 years.
Patient 15	c.3610C > T (p.Arg1204*)	M	4 years 6 months	+	+	+	−	−	−	N/A	N/A	N/A	Normal brain MRI	−	N/A
Patient 16 *(mother of patient 15)*	c.3610C > T (p.Arg1204*)	F	N/A	N/A	N/A	N/A	N/A	N/A	N/A	N/A	N/A	N/A	N/A	N/A	N/A
Patient 17	c.4555C > T (p.Arg1519*)	F	9 years	+ (mild)	+	+	−	+	+	Lateral flaring of eyebrows, narrow palpebral fissures, prominent ears and cheekbones, small mouth, micrognathia	Transient strabismus when tired	Overlapping second toe on left foot, small sandal gap deformity of both feet	Normal brain MRI	Epileptic seizures treated with levetiracetam	Infantile autism, hyperhidrosis. IQ 70.
Patient 18	c.5936‐1G > A (p.Thr1980Glufs*25)	M	8 years 8 months	+ (mild)	+	+	−	N/A	N/A	Prominent forehead, prominent nasal bridge, mild malar hypoplasia, medial flaring of the eyebrows, telecanthus, mild retrognathia	None	Cutaneous syndactyly of hands (3–4) and feet (2–3) ‐ likely autosomal dominant syndactyly based on family history; mild pes planus, bulbous halluces	N/A	N/A	Severe feeding problems requiring gastrostomy; congenitally corrected transposition of the great arteries. Mild to moderate intellectual disability (IQ 57).
Patient 19	c.6078dupT (p.Ala2027Cysfs*2)	M	12 years	+	+	+	−	N/A	N/A	Micrognathia, dental crowding, midface hypoplasia, upslanting palpebral fissures	Left eye ptosis, limited peripheral vision, 20/25 vision	Leg length discrepancy, mild scoliosis, 2–3 syndactyly bilateral, tooth eruption at 2–4 m	Normal brain MRI	N/A	Bilateral polycystic kidneys diagnosed prenatally, constipation, unilateral cryptorchidism (left)
Patient 20	c.6259+3_6259 + 4delinsG	F	9 years	+ (mild)	+	+	+	N/A	N/A	Prominent long nose, micrognathia, lateral flaring of eyebrows	Unilateral exotropia	Thoracic kyphosis, sandal gap both feet, 2–3 syndactyly of both feet, broad great toes, long slender fingers	N/A	N/A	Hypermobility
Patient 21	c.7521_7524dupATCT (p.Leu2509Ilefs*21)	M	10 years	+ (mild)	+	+	+	N/A	N/A	Narrow face shape with mild retruded midface and slightly prominent metopic suture, subtle upslant of eyes with mild ptosis; epicanthal folds present, flat philtrum with thin lips. First toe bilaterally is slightly laterally deviated and a little bit broad	Stable hyperopia and alternating exotropia, wears glasses	Thoracolumbar scoliosis (improved ‐ previously required bracing) and leg length discrepancy; butterfly vertebra at T12 and questionable fusion of right posterolateral aspects of T5 and T6 vertebral bodies	Mild confluent T2/FLAIR hyperintense signal in the periatrial white matter, with associated slight prominence of adjacent atrium of lateral ventricles; probably represents sequelae of old insult	Complex focal seizures with impaired awareness of frontal lobe origin beginning at age 7 y, controlled by medication with recent vagal nerve stimulator placement	Significant failure to thrive and small for gestational age; severe bleeding problem after circumcision. Delays and mild intellectual disability (IQ of 52); spoke first words at 4 years, spoken language level is not age appropriate; walked at 16 months.
Patient 22^a^	c.7875+3559_7876‐2789del and t(1;17)(q24.3;q24.2)	M	8 years	+	+	+	+	N/A	N/A	Prominent mid‐forehead, craniofacial asymmetry, asymmetric face, triangular shape, asymmetric zygomatic region, hypertelorism, proptosis, lateral flaring of the eyebrows, broad eyebrows, broad palpebral fissures, long eyelashes, long nasal bridge, prominent nose, broad nose tip, thin upper lip, micrognathia, microglossia, microgenia, retrogenia, high palate, crowded small teeth, protruding ears, different size of ears, asymmetric location of ears, large helices of both ears, low‐set or posteriorly rotated ears, prominent ears, anteriorly rotated left ear axis, broad tragus, small lobes	Myopia, strabismus, absence of binocular single vision and stereopsis	Scoliosis, narrowing intervertebral foramen and canal of lumbosacral part of spine L4‐L5‐S1, fifth finger clinodactyly, shorter phalanges of fifth fingers and halluces, bilateral partial 2–3 syndactyly, sandal gap deformities of both feet	Thickening of the bone of the skull's vault, the ssella turcica extended with a flat bottom, the anterior pituitary is uniformly enhanced after gadolinium administration, but there is decreased height of the gland (4 mm), upper surface of the anterior pituitary is flat, the L‐basilar artery is tortuous and elongated, but it is not widened; adheres to the brainstem with a small degree of brainstem compression, cortical and subcortical atrophy with widening of intracranial fluid spaces	N/A	Feeding difficulties, hyperhidrosis, delayed psychomotor development, deep feeling of both feet is incorrect, left ventricular failure, diabetes, hypercholesterolemia, decreased estradiol. Difficulties with comprehending the meaning of text, disturbances with recognition of mistakes in written text, difficulties with subtraction and operations performed on numbers of higher values; difficulties remembering items directly, learning difficulties, dyslexia and dysgraphia due to a defect of phonemic audition.
Patient 23	c.8081G > C (p.Arg2694Thr)	M	9 years	−	+ (mild)	−	−	N/A	N/A	N/A	None	None	N/A	N/A	Horseshoe kidney, VSD closed spontaneously
Patient 24	c.(7875 + 1_7876–1)_(8209+1_8210‐1)del	F	12 years 3 months	+	+	−	−	N/A	N/A	Mild facial dysmorphism—short palpebral fissures, thin upper lip lateral flaring of eyebrows	No	None	Normal brain MRI	EEG abnormalities, focal temporoparietal left, but no clinical seizures	Mild intellectual disability; IQ‐Test 2016:69 (HAWIK IV); First words at 2 years 3 months; still speaks in simple sentences. History of anxiety.
Patient 25	c.8210+6_8210+8del	F	2 years	+	+	+	+	N/A	N/A	Low set ears, slightly upslanting palpebral fissures, epicanthal folds, 3 small irregular café au lait macules, unilateral single palmar crease	Bilateral persistent pupillary membrane, unilateral iris coloboma, unilateral subtle subluxated lens	Sandal gap deformities	Normal brain MRI	N/A	Hypoplastic index finger nail/ nail bed. Developmental delay though no intellectual disability.
Patient 26	c.8278G > T (p.Glu2760*)	M	11 years	+	+	+	+ (mild)	N/A	N/A	Preauricular chondroma (unilateral), 3 café au lait macules	Exotropia of left eye, hypermetropia	N/A	N/A	EEG at 6y with spikes in right frontal region, enhanced by photic stimulation and sleep—no clinical seizures	Mild to moderate ID, did not meet criteria for autism although had stereotypies; first words at 3years; held head up at 9 monthsonths, sat at 15 months, walked at 20 months.

Abbreviation: N/A, not available or not reported.

^a^ Previously reported (Midro et al., 1993, 2019).

#### 3.3.3 Dysmorphic features and other findings

**FIGURE 2:**
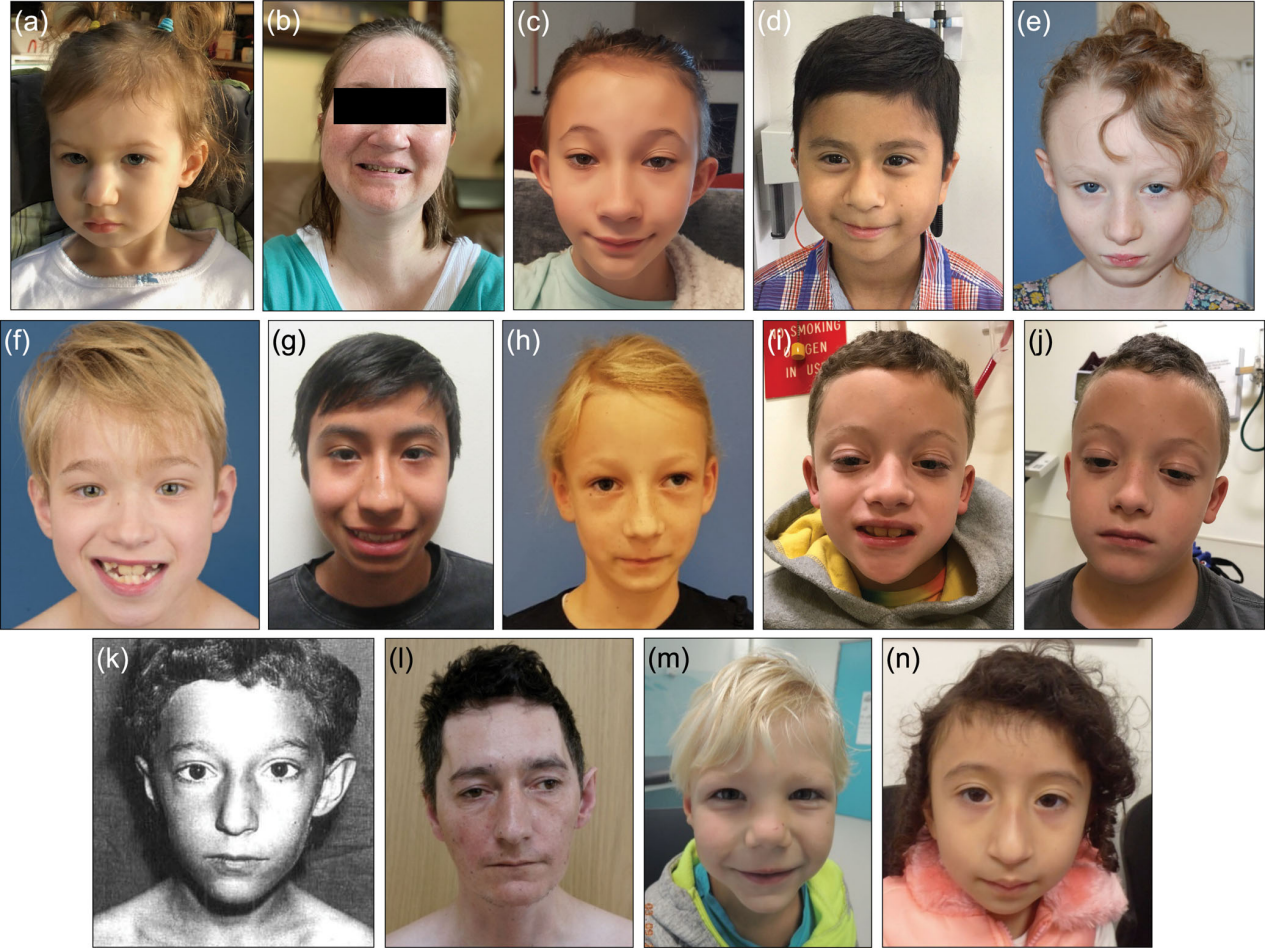
(a) Patient 1, (b) Patient 2, (c) Patient 5, (d) Patient 12, (e) Patient 17, (f) Patient 18, (g) Patient 19, (h) Patient 20, (i) Patient 21 at age 9 years 10 months, (j) Patient 21 at age 10 years 3 months, (k) Patient 22 at age 8 years(Midro et al., 1993), (l) Patient 22 at age 35 years (Midro et al., 2019), (m) Patient 23, (n) Subject 5 from Stankiewicz et al., 2017 (Stankiewicz et al., 2017). Note the presence of prominent nasal ridge (a, b, c, e, g, h, i, k, l, n), bulbous nasal tip (a, d, h, k, m, n), and pointed chin (a, c, d, e, f, i, k, m, n) [Color figure can be viewed at wileyonlinelibrary.com]

As noted previously (Stankiewicz et al., 2017), mild dysmorphic features were identified in all available subjects (20/20 individuals) (Figure 2). Commonly identified features included a number of nasal abnormalities (prominent nasal tip, bulbous nasal tip, and prominent nasal bridge) in 9/20 (45%) individuals (Table 2). Interestingly, 13 individuals demonstrated mild ophthalmologic abnormalities. These included the finding of exotropia in six individuals, strabismus in two individuals, and confirmed myopia in three individuals.

**FIGURE 3:**
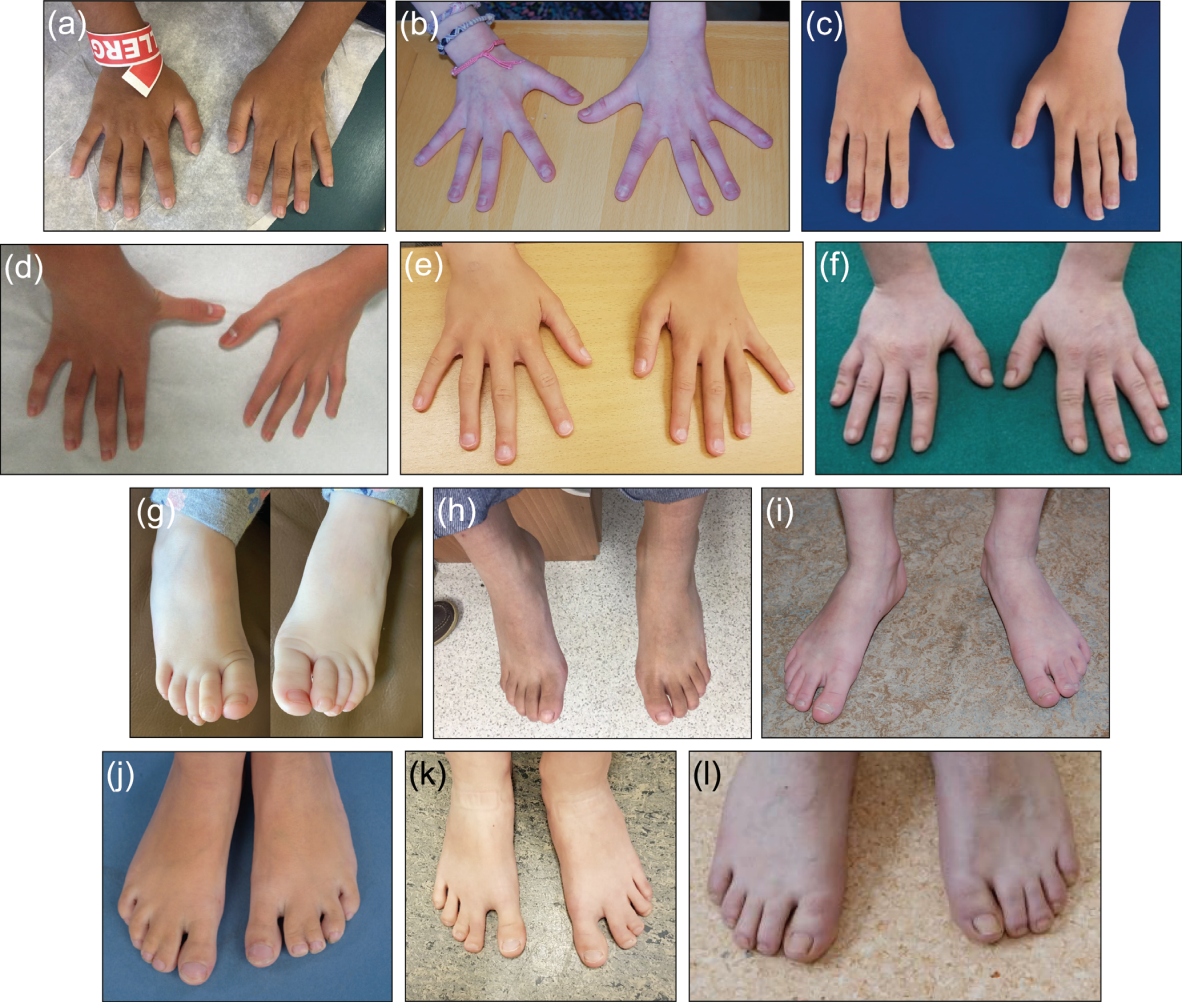
(a) Patient 12, (b) Patient 17, (c) Patient 18, (d) Patient 19, (e) Patient 20, (f) Patient 22, (g) Patient 1, (h) Patient 12, (i) Patient 17, (j) Patient 18, (k) Patient 20, (l) Patient 22. Note the presence of fifth finger clinodactyly (d), sandal gap deformity (i, k) and cutaneous syndactyly (j, k) [Color figure can be viewed at wileyonlinelibrary.com]

We identified skeletal abnormalities in a total of 13 individuals. These included the presence of scoliosis in five individuals (5/13, 38.5%), along with kyphotic deformities in two individuals (2/13, 15%), and delayed bone age in two individuals (2/13, 15%). We also identified five cases of cutaneous syndactyly (2–3), along with five individuals with sandal‐gap anomalies, and limb‐length discrepancies in two individuals (Figure 3).

## 4 DISCUSSION

The rapid advancements of genomic technologies have led to an almost continuous stream of new diseases and syndromes based on a common defect gene. This “genotype‐first” approach has gained prominence, particularly in complex diseases like autism spectrum disorders or DD where affected individuals often display a range of nonspecific symptoms and manifestations (Stessman et al., 2014). There still remains, however, a significant role for careful and thoughtful descriptions of clinical phenotypes in a given disease, particularly in Mendelian or single‐gene disorders. Here, we have sought to further characterize and clarify the clinical phenotype of individuals with *BPTF*‐related NEDDFL. Our analyses indicate that the spectrum of findings in affected individuals is wider than previously noted. Indeed, BPTF appears to have effects on almost every part of the body as befitting its presumed critical role in human development.

In addition to DDs, microcephaly, and distal limb defects, our cohort exhibited an increased frequency of ophthalmologic abnormalities, poor weight gain/poor feeding, and cutaneous syndactyly of the toes. Prior to our study, seizures had never been reported in individuals with NEDDFL. Here, however, we identified six individuals with a history of clinical seizures and/or abnormal electroencephalograms (manuscript in preparation). This along with the identification of five individuals with abnormal MRI findings further supports the critical role of *BPTF* in neurodevelopment and neural regulation. Given these observations, providers should monitor for any signs of seizures or spells and have a low threshold for obtaining an EEG or MRI in patients with NEDDFL.

Similarly, we also identified the presence of skeletal abnormalities in 50% (13/26) of individuals, including patients with scoliosis and spinal anomalies, delayed bone age, and limb‐length discrepancies. The mechanism for these abnormalities is less clear, though it is presumed that they are also derived from abnormal signaling during embryogenesis similar to the distal limb defects commonly described in these patients. While it is unclear how significant the degree of scoliosis was within individuals, careful examination and monitoring for this clinical finding are also recommended.

Cardiac abnormalities remained rare within our cohort, though one individual was born with congenitally corrected transposition of the great arteries, whereas another patient was reported to have left ventricular failure.

Other rare findings in the cohort included a history of recurrent fevers, oral ulcers, and skin rashes in one individual and hyperhidrosis in two individuals. One patient (Patient 21) was reported to have severe bleeding following his circumcision, whereas another (Patient 9) had frequent infections requiring intravenous immunoglobulin treatments until the age of 4 years. Two patients had dyslipidemia (Patient 5 with mildly elevated LDL and low HDL cholesterol levels, and Patient 22 with reported hypercholesterolemia), whereas one patient (Patient 8) had very little subcutaneous fat.

Our study has also uncovered four cases of inherited *BPTF* variants, providing some insight into the penetrance and expressivity of NEDDFL. Though complete clinical data were not available for all four parents, we were able to obtain limited information on three of these individuals. Patient 13, the father of Patient 11, had a history of DD and delays in school; however, since leaving school, he has attained employment and presumably functions independently in society as an adult. No mention was made of this patient or Patient 16 (the affected mother of Patient 15) as having microcephaly; however, it is unclear whether this was ever clinically ascertained. Patient 10, the mother of Patient 9, was found to have microcephaly though she does not have a history of reported delays or ID. Interestingly, Patient 2 (the mother of Patient 1) was described by her own mother as being small for gestational age, having a “small head” as a child and exhibiting significant feeding difficulties. She had substantial learning disabilities in school and though she is able to function reasonably independently as an adult, she continues to live with her own mother who assists her with childcare and some day‐to‐day activities. The histories reported in these patients and the presence of inherited pathogenic variants in *BPTF* do suggest a pattern of variable expressivity in NEDDFL. It is possible that this is related to patients' genotypes; however, additional clinical information is needed to definitively characterize these relationships. Given its role in chromatin remodeling, it is likely that the severity of NEDDFL is heavily influenced by additional epigenetic factors.

The present study has some inherent limitations. Subjects in our study have been recruited from different clinical settings and in several different countries. It is thus unsurprising that the availability of records and willingness of families to participate varied significantly. These factors may, therefore, result in an underestimation of the frequency of some of the clinical features, particularly as some patients have been lost to follow‐up since initial enrollment. Additionally, because of the different molecular diagnostic techniques used (i.e., exome sequencing, panel testing, etc.) and differences in coverage, it is possible that some patients may harbor variants in other neurodevelopmental genes, which may be modifying their presentations. Finally, as the recognition of this disorder increases, it is anticipated that the finding of missense variants in *BPTF* may present a diagnostic challenge for practitioners, particularly in individuals with mild phenotypes. Though there is not a well‐validated functional assay for the disorder at this time, the use of RNA‐sequencing or proteomics may have some utility in such cases.

The present study does, however, add significantly to the clinical description of NEDDFL and more than doubles the number of affected individuals in the literature. The clinical and molecular information gathered may allow for increased recognition of this disorder on the part of practitioners and shorten the diagnostic odyssey experienced by many patients. Also, the descriptions here may provide additional diagnostic, prognostic, and management information for both providers and caregivers.

## CONFLICT OF INTEREST

The authors declare no conflict of interest.

## AUTHOR CONTRIBUTIONS

Kevin E. Glinton collected data, analyzed and interpreted the results, and wrote the manuscript. Anna C. E. Hurst, Kevin M. Bowling, Ingrid Cristian, Devon Haynes, Dusit Adstamongkonkul, Oskar Schnappauf, David B. Beck, Carole Brewer, Aditi Shah Parikh, Deepali N. Shinde, Alan Donaldson, Ariel Brautbar, Saskia Koene, Arie van Haeringen, Amélie Piton, Yline Capri, Margherita Furlan, Elena Gardella, Rikke Steensbjerre Moller, Irma van de Beek, Linda Zuurbier, Phillis Lakeman, Allan Bayat, Julian Martinez, Rebecca Signer, Pernille M. Torring, Morten Buch Engelund, Karen W. Gripp, Louise Amlie‐Wolf, Lindsay B. Henderson, Alina T. Midro, Eugeniusz Tarasów, Beata Stasiewicz‐Jarocka, Diana Moskal‐Jasinska, Paul Vos, Felix Boschann, Corinna Stoltenburg, Oliver Puk, Inger‐Lise Mero, Kristine Lossius, Cyril Mignot, Boris Keren, Johanna C. Acosta Guio, Ignacio Briceño, and Alberto Gomez performed the clinical evaluation. Yaping Yang interpreted the results and critically revised the final version of the article. Pawel Stankiewicz designed the study concept, coordinated and supervised the work, and critically revised the article.

## Supporting information


**Appendix** **S1:** Supporting information

## Data Availability

The data that support the findings of this study are available from the corresponding author upon reasonable request.
