# Supplementary material for: Phenotypic expansion of the BPTF‐related neurodevelopmental disorder with dysmorphic facies and distal limb anomalies
Source: Am J Med Genet A. 2021 Jan 31;185(5):1366–78. doi: 10.1002/ajmg.a.62102 (PMC8048530; doi:10.1002/ajmg.a.62102)
Supplement: Supplementary file 1 — Appendix S1: Supporting information [file AJMG-185-1366-s001.docx]

**Supplementary Table 1**: Molecular Testing results of patients with *BPTF* variants (isoform NM_004459.6).

| **Patient** | **Variant** | **Exon (or Intron)** | **Variant Type** | **Inheritance** | **Molecular Testing** | **ACMG Classification** | **Other variants** |
| --- | --- | --- | --- | --- | --- | --- | --- |
| 1 | c.209dupG (p.Ser71Glnfs*3) | 1 | Frameshift | Maternal | Exome Sequencing (Trio) | Likely pathogenic (PVS1+PM2) | No other variants reported |
| 2 *(mother of Patient 1)* | c.209dupG (p.Ser71Glnfs*3) | 1 | Frameshift | N/A | Exome Sequencing (Trio) | Likely pathogenic (PVS1+PM2) | No other variants reported |
| 3 | c.255delC (p.Ser86Alafs*151) | 1 | Frameshift | de novo | Genome Sequencing as part of a research study, the Alabama Genomic Health Initiative (AGHI), funded by the state of Alabama. | Pathogenic (PVS1+PS2+PM2+PP5) | No other variants reported |
| 4 | c.255delC (p.Ser86Alafs*151) | 1 | Frameshift | de novo | Sequence Analysis – Autism/ID Xpanded Panel by GeneDx | Pathogenic (PVS1+PS2+PM2+PP5) | Heterozygous variant of uncertain significance in *FAT3* |
| 5 | c.255dupC (p.Ser86Glnfs*43) | 1 | Frameshift | de novo | Sequence Analysis – Autism/ID Xpanded Panel by GeneDx | Pathogenic (PSV1+PS2) | No other variants reported |
| 6 | c.1282G>T (p.Glu428*) | 2 | Nonsense | de novo | Exome Sequencing, Sanger sequencing and CLIA Sanger for confirmation. | Pathogenic (PVS1+PS2+PM2) | No other variants reported |
| 7 | c.1607_1620del (p.Asp536Glyfs*5) | 3 | Frameshift | N/A | Duo Exome Sequencing through Deciphering Developmental Disorders Study.(father not available). Confirmed by targeted Sanger sequencing in NHS accredited lab | Likely pathogenic (PVS1+PM2) | Maternally inherited hemizygous missense variant of uncertain significance in *HCFC1* c.4340C>T (NM_005334.2) |
| 8 | c.2724_2727del (p.Thr909Serfs*4) | 8 | Frameshift | de novo | Exome Sequencing | Pathogenic (PVS1+PS2+PM2) | No other variants reported |
| 9 | c.2921+1G>C | Intron 9 | Splicing | Maternal | WGS as part of 100,000 genomes | Likely pathogenic (PVS1+PM2) | No other variants reported |
| 10 *(mother of Patient 9)* | c.2921+1G>C | Intron 9 | Splicing | N/A | WGS as part of 100,000 genomes | Likely pathogenic (PVS1+PM2) | No other variants reported |
| 11 | c.3085delA (p.Thr1029Glnfs*27) | 11 | Frameshift | de novo | Exome Sequencing | Pathogenic (PVS1+PS2+PM2) | 1.393 Mb Microduplication of uncertain significance at 16p13 |
| 12 | c.3210_3221del (p.Asn1071Glu1074del) | 12 | In-frame Deletion | Paternal | Genome Sequencing as part of a research study, the Alabama Genomic Health Initiative (AGHI), funded by the state of Alabama. | VUS  (PM2+ PM4) | No other variants reported |
| 13 *(father of Patient 12)* | c.3210_3221del (p.Asn1071Glu1074del) | 12 | In-frame Deletion | N/A | Genome Sequencing as part of a research study, the Alabama Genomic Health Initiative (AGHI), funded by the state of Alabama. | VUS  (PM2+PM4) | No other variants reported |
| 14 | c.3233_3237del (p.Arg1078Metfs*13) | 12 | Frameshift | N/A | Exome Sequencing | Likely pathogenic (PVS1+PM2) | Maternal 17q22 duplication of uncertain significance |
| 15 | c.3610C>T (p.Arg1204*) | 13 | Nonsense | Maternal | Panel sequencing (456 genes involved in NDD) | Likely pathogenic (PVS1+PM2) | No other variants reported |
| 16 *(mother of patient 15)* | c.3610C>T (p.Arg1204*) | 13 | Nonsense | N/A | Sanger Sequencing | Likely pathogenic (PVS1+PM2) | N/A |
| 17 | c.4555C>T (p.Arg1519*) | 13 | Nonsense | de novo | Array CGH, EPIDASD Panel by Amplexa Genetics | Pathogenic (PVS1+PS2+PM2) | Heterozygous variants of uncertain significance in *COL6A2* and *ACSL4*. Likely benign rare duplication of 12p13, inherited from the father |
| 18 | c.5936-1G>A (p.Thr1980Glufs*25) | Intron 15 | Splicing | de novo | Trio Exome Sequencing. RNA-analysis showed aberrant splicing; an alternative splice site in exon 16 is used instead of the original donor splice site of intron 15. This leads to a deletion of 17 nucleotides of exon 16 and a frameshift, leading to a premature stop codon | Pathogenic (PVS1+PS2+PM2) | No other variants reported |
| 19 | c.6078dupT (p.Ala2027Cysfs*2) | 16 | Frameshift | de novo | Trio Exome Sequencing | Pathogenic (PVS1+PS2+PM2) | No other variants reported |
| 20 | c.6259+3_6259+4delinsG | Intron 18 | Splicing | de novo | Trio Exome Sequencing (Illumina NextSeq550, NimbleGen SeqCap EZ MedExome). | Likely pathogenic (PS2+PM2) | No other variants reported |
| 21 | c.7521_7524dupATCT (p.Leu2509Ilefs*21) | 23 | Frameshift | de novo | Exome Sequencing | Pathogenic (PVS1+PS2+PM2) | c.992 C>T (p.Pro331Leu) in exon 8 in the *F10* gene (NM_000504.3); VUS |
| 22† | c.7875+3559_7876-2789del and t(1;17)(q24.3;q24.2) | Intron 25 | Gene Disruption/Deletion | de novo | Chromosome analysis, Fluorescence in situ hybridization, Whole Genome Sequencing, Sanger Sequencing | Pathogenic  (PVS1+PS2) | N/A |
| 23 | c.8081G>C (p.Arg2694Thr) | 26 | Missense | N/A^*^ | Exome and Sanger Sequencing | VUS  (PM2+PM6+PP3) | No other variants reported |
| 24 | c.(7875+1_7876-1)_(8209+1_8210-1)del | 26 | Exon Deletion, Frameshift | de novo | Trio Exome Sequencing, Deletion of Exon26 confirmed by qPCR | Pathogenic  (PVS1+PS2) | No other variants reported |
| 25 | c.8210+6_8210+8del | Intron 26 | In-frame Deletion | de novo | Trio Exome Sequencing applying a ID-panel (resembling DDD2) | Likely pathogenic (PS2+PM2) | N/A |
| 26 | c.8278G>T (p.Glu2760*) | 27 | Nonsense | de novo | Trio Exome Sequencing | Pathogenic (PVS1+PS2+PM2) | Compound heterozygous variants in *PCNX2*, *MICALL2*, *DNAH11*; X-linked variants in *MAP7D2*, *ZCCHC13*, *EGFL6* |

N/A – Not available

*Parental samples were negative by Sanger sequencing. Paternity and maternity were not confirmed.

†Previously reported (Midro et al. 1993, 2019)
